# Forty-four Years of the UK Prospective Diabetes Study: Legacy Effect and Beyond

**DOI:** 10.17925/EE.2025.21.1.8

**Published:** 2024-11-20

**Authors:** Saptarshi Bhattacharya, Sanjay Kalra, Lakshmi Nagendra, Deep Dutta

**Affiliations:** 1. Department of Endocrinology, Indraprastha Apollo Hospitals, New Delhi, India; 2. Department of Endocrinology, Bharti Hospital, Karnal, Haryana, India; 3. Department of Endocrinology, JSS Medical College, JSS Academy of Higher Education and Research, Mysore, India; 4. 4. Department of Endocrinology, Center for Endocrinology, Diabetes, Arthritis & Rheumatism, Dwarka, New Delhi, India

**Keywords:** Intensive glycaemic control, legacy effect, macrovascular complication, metabolic memory, metformin, type 2 diabetes

## Abstract

The results of the third phase of the UK Prospective Diabetes Study, recently published in *The Lancet*, reinforce the crucial role of early intensive glycaemic control in protection against micro- and macrovascular complications of diabetes. The benefits persist into the third decade after the completion of the trial, long after the difference in glycated haemoglobin between the intensive and standard arms has disappeared. This ‘legacy effect’ emphasizes the need for early diagnosis and aggressive management of diabetes from the time of its identification. It also validates the long-term safety and efficacy of conventional agents such as metformin and sulfonylureas. Understanding the mechanisms behind the ‘legacy effect’ could help target pathways that lead to the development of complications.

Very few trials in the history of medical science have altered the treatment landscape as profoundly as the UK Prospective Diabetes Study (UKPDS). Even 44 years after its inception, the trial and poststudy follow- up findings continue to fascinate and enlighten the medical community. The study was conceived at a time when there was uncertainty about whether treating hyperglycaemia actually improved outcomes. Indeed, results from the University Group Diabetes Program suggested that treatment with tolbutamide and phenformin was more harmful than beneficial.^1^

The primary objective of the original study was to ascertain whether intensive glycaemic control reduced morbidity and mortality in people with recently diagnosed type 2 diabetes (T2D) compared with standard management. Another important aim was to determine whether diet alone, sulfonylureas, insulin or metformin offered a specific advantage over the others.

## The three phases of the UK Prospective Diabetes Study

The study commenced in 1977 and included 600 patients in a pilot phase in five centres.^2^ By 1987, the study had expanded to 23 centres (15 centres in Glucose Study I and 8 in Glucose Study II), enrolling a total of 5,102 participants.^3^ The intervention part of the trial lasted up to 20 years. The second phase involved a 10-year post-study follow-up, during which participants attended the clinic for the first 5 years.^4^ The third period, from 2007 to 2021, reflects information accessed from the National Health Service administrative data. The UKPDS provides 80,724 person-years of follow-up data in the three phases combined and qualifies as one of the longest trials in the history of medical science.^5^

## Key findings in the first two phases

The first phase of the UKPDS demonstrated that strict glycaemic control with sulfonylureas or insulin significantly decreased the risk of microvascular but not macrovascular complications.^6^ Additionally, those who were overweight and randomized to the metformin arm had a reduced risk for any diabetes-related endpoint, diabetes-related death and all-cause mortality compared with those allocated to conventional therapy. During the 10 years of follow-up, the median glycated haemoglobin (HbA1c) was 7.4% in the metformin group and 8.0% in the conventional treatment group. Patients assigned to intensive control with sulfonylureas or insulin had similar HbA1c levels to those in the metformin group. Metformin outperformed chlorpropamide, glibenclamide or insulin for most diabetes-related outcomes.^7^ The UKPDS results led to the acceptance of intensive glycaemic control as the standard management of T2D, with metformin as the first-l ine agent.

The second phase of the observational 10- year post- trial monitoring, with no glycaemic difference between the two arms, revealed some exciting outcomes. Not only did the microvascular protection persist after 10 years, but there was also an emergent risk reduction for myocardial infarction (MI) and all-cause mortality in the intensive arm. The continued long-term benefits accrued from early intensive glycaemic control are described as the ‘legacy effect’ or ‘metabolic karma’.^4,8,9^

## The third phase results from the 44-year follow-up

The recent publication on the third phase of the UKPDS follow-up data in *The Lancet* further highlights the long-term benefits of intensive diabetes treatment.^5^ With individual follow-up ranging from 0 to 42 years and a median of 17.5 years, the study reveals that participants initially randomized to intensive insulin or sulfonylurea therapy had a 17% reduced risk of MI, a 26% reduced risk of microvascular events and a 10% reduced risk of all-cause mortality compared with those allocated to conventional therapy. In the metformin-i ntensive arm of the study, there was an 18% reduction in any diabetes-related endpoint, a 31% reduction in MI and a 20% reduction in all-cause mortality compared with controls.^5^ However, microvascular benefit was not maintained in the metformin arm. These results demonstrate that the ‘legacy effect’ persists for an even longer duration than initially demonstrated. Early intensive glycaemic control, even with conventional medications such as metformin and sulfonylureas, continues to prevent complications and enhance survival.

## Comparison of the UK Prospective Diabetes Study with other trials

Three trials assessing the effects of intensive glycaemic control were published around the same time as the UKPDS 10-year follow-up study.^4^ These were the ACCORD (Action to Control Cardiovascular Risk in Diabetes; ClinicalTrials. gov Identifier: NCT00000620) study , the ADVANCE (Action in Diabetes and Vascular Disease: Preterax and Diamicron Modified Release Controlled Evaluation; ClinicalTrials. gov Identifier: NCT00145925) trial and the VADT (Veterans Affairs Diabetes Trial; CSP #465 – Glycemic Control and Complications in Diabetes Mellitus Type 2; ClinicalTrials. gov Identifier: NCT00032487) trial.^10–12^

Intensive glycaemic control did not lead to a significant reduction in cardiovascular (CV) events in the ADVANCE trial and VADT, while the ACCORD trial resulted in an increased overall and CV mortality. In contrast to the UKPDS, the three trials had participants with a longer duration of diabetes with a higher baseline prevalence of risk factors and diabetes complications. The aggressive management initiated early in the UKPDS, compared with the later initiation for advanced diabetes in the other three trials, is the likely explanation for these contrasting findings. Although the overall event rate was low in the ACCORD trial, the increased mortality could be due to a rapid reduction and maintenance of low HbA1c levels, combined with the effects of severe hypoglycaemia. As expected, all these trials of intensive glycaemic control resulted in a consistent decline in microvascular complications.^4,10–12^

The CV outcome trials (CVOTs), conducted as per the US Food and Drug Administration’s requirement for developing a new anti-diabetic drug, cannot be compared with the UKPDS. The CVOTs were mostly phase IV event-driven trials targeting over 600 primary endpoint events designed to rule out CV risk with statistical confidence (a hazard ratio with an upper confidence limit of ≥1.3 for a 3-point major adverse cardiovascular events). The study populations included individuals with multiple CV risk factors or established CV disease to ensure the timely completion of the trials.^13^

It would be interesting to observe the findings of a trial conducted along the lines of the UKPDS, with newer drugs with CV superiority. In addition, the long-term follow-up results of newer drugs, which have shown benefits in the CVOTs, would be noteworthy. We should also keep in mind that metabolic memory can extend beyond hyperglycaemia and multifactorial target-driven therapy, as demonstrated in Steno-2 (Intensified Multifactorial Intervention in Patients With Type 2 Diabetes and Microalbuminuria; ClinicalTrials. gov Identifier: NCT00320008), is most likely to produce maximum benefit.^14^

## Possible explanation of the legacy effect

Though the exact mechanism is not yet understood, several adaptations could account for the legacy effect. Hyperglycaemia-i nduced epigenetic variations, such as histone modifications, DNA methylation and noncoding microRNAs, trigger the formation of pro-i nflammatory genes that persist even after the removal of the hyperglycaemic insult. The state of low-grade chronic inflammation resulting from the continued expression of pro-i nflammatory genes could be responsible for metabolic memory.^15^

Mitochondrial superoxide generation and the subsequent alterations in cellular and metabolic processes are also anticipated to play a major causative role in driving long-term complications. The production of reactive oxygen species induces the formation of receptors for advanced glycation end-products (RAGEs). The overproduction of superoxide ions and RAGEs persists even after the normalization of glucose levels.^16,17^ Additionally, impaired healing of microvasculature in response to ischaemia can induce endothelial dysfunction and continued micro- and macrovascular damage.^18^

## Key lessons from the UK Prospective Diabetes Study

The findings of the UKPDS reinforce the strategy of ‘hitting early’ and ‘hitting hard’ right after the diagnosis of T2D. If this window of opportunity is lost, the risk of both short- and long-term complications is considerably aggravated. Timely diagnosis and early intensive glycaemic control can have a long-l asting impact on health, quality of life and survival.

The availability of newer agents that offer CV protection without increasing the risk of hypoglycaemia further expands the possibilities for safely and effectively achieving this target. However, the role of older drugs such as metformin and sulfonylureas should not be overlooked. The findings from 44 years of the UKPDS strongly corroborate their long-term safety and efficacy. The advantages of metformin probably extend beyond its glycaemic efficacy, as its positive impact persists despite only a marginal difference in HbA1c between the two arms in the trial.

## Conclusion

An early, multipronged and aggressive approach to managing T2D creates the ideal metabolic memory and provides the best solution to this chronically progressive condition. The personalized use of newer therapies with CV superiority, alongside conventional treatments such as metformin and sulfonylureas, is recommended to optimize outcomes. Early intensive glycaemic control has long-l asting benefits for complications and survival, and such an approach should be the norm for treating newly diagnosed T2D. Additionally, researching novel therapies that target epigenetic alterations to reverse detrimental metabolic memory could offer a strategy to prevent complications.
